# The Distribution of SIgA and IgG Antibody-Secreting Cells in the Small Intestine of Bactrian Camels (*Camelus bactrianus*) of Different Ages

**DOI:** 10.1371/journal.pone.0156635

**Published:** 2016-06-01

**Authors:** Wang-Dong Zhang, Wen-Hui Wang, Shuai Jia

**Affiliations:** College of Veterinary Medicine, Gansu Agricultural University, Lanzhou, Gansu, China; University of Missouri-Kansas City, UNITED STATES

## Abstract

Secretory immunoglobulin A (SIgA) and immunoglobulin G (IgG) antibody-secreting cells (ASCs) are two important cell types in the mucosal immune system. This study aimed to explore the distribution of these ASC populations in the small intestine of Bactrian camels of different ages. Twenty-four Alashan Bactrian camels were divided into the following four age groups: young (1–2 years), pubertal (3–5 years), middle-aged (6–16 years) and old (17–20 years). SIgA and IgG ASCs in the intestinal mucosa lamina propria (LP) were observed and analyzed using immunohistochemcal techniques. The results from all age groups show that both SIgA and IgG ASCs were diffusely distributed in the intestinal LP, and some cells aggregated around the crypts. Moreover, the densities of the two ASC populations gradually increased from the duodenum to the jejunum and then decreased in the ileum. Meanwhile, there were more SIgA ASCs than IgG ASCs in the duodenum, jejunum, and ileum, and these differences were significant in the young and pubertal groups (*P*<0.05). In addition, the SIgA and IgG ASC densities increased from the young to the pubertal period, peaked at puberty, and then gradually decreased with age. The results demonstrate that the SIgA and IgG ASC distributions help to form two immunoglobulin barriers in the intestinal mucosa to provide full protection, helping to maintain homeostasis. These findings also underscore the importance of researching the development and degeneration of intestinal mucosal immunity in Bactrian camels.

## Introduction

The mammalian intestine harbors a complex microbial community that is established after birth [1–3]. Microbes increase the risk of acute intestinal inflammation [4,5], but, also provide nutrients and energy for the host [6–9]. However, these microbes establish symbiotic relationships with their hosts because the gastrointestinal mucosal immune system can accurately distinguish pathogenic and commensal microorganisms and can induce immune responses accordingly [10,11]. Therefore, the gastrointestinal mucosal immune system is one of the most important components of the body’s immune system.

Secretory IgA (SIgA) is one of the most important effector molecules in the gastrointestinal immune system because it constitutes the first-line immunological barrier against pathogens; it modulates immune exclusion [12–14], regulates the intestinal microecology [15], induces immune tolerance [16–18], and inhibits inflammation and allergic reactions, as well as performing other functions [19]. However, when this barrier is destroyed, invasive pathogenic microorganisms can cross the epithelial border. Subsequently, another important effector molecule, IgG, rapidly recruits phagocytic innate immune cells (granulocytes, monocytes) through the activation of an inflammatory reaction. With the help of IgG, phagocytic cells eliminate the invading bacteria in a matter of hours [20]. Therefore, IgG provides a second line of defense that controls microbial dissemination by eliciting a robust inflammatory reaction.

Several previous studies have shown that the proportions of antibody-secreting cells (ASCs) differ among mucosal regions. For example, SIgA and IgG ASCs account for approximately 79% and 3–4%, respectively, of the cells in the intestinal mucosa of normal adult human. However, these ASC populations represent approximately 69% and 17% of the cells in the nasal mucosa and 76% and 13% in the gastric mucosa, respectively [21,22]. Moreover, studies have described unique characteristics related to the gastrointestinal mucosal immune system of Bactrian camels (*Camelus bactrianus*), an economically important livestock species in northwest China. Wen-hui Wang et al. found an area with a triangular, band-like aggregated lymphoid nodule in the cardiac gland region of the third compartment of the Bactrian camel’s stomach [23–25]. Such a structure has not been reported in other animals, including dromedary camels (*Camelus dromedarius*) [26]. The morphology of Payer’s patch (PP) in the small intestine of Bactrian camels is diverse and includes nodular, faviform and scrotiform subtypes [27,28]. Moreover, C.Hamers-Casterman et al. reported that Camelidae IgG2 and IgG3 are heavy chain antibodies (HCAbs) [29,30]. Unlike general IgG antibodies, the structure of HCAbs is unique and naturally devoid of light chain, resulting in an antigen binding site with only a single domain [31].

However, few reports have examined the distribution of SIgA and IgG ASCs in the digestive tract of Bactrian camels or how these cell populations change with age. In this study, the distribution characteristics, densities and age-related alterations of SIgA and IgG ASCs in the small intestinal lamina propria (LP) of Bactrian camels were observed and analyzed. These data provide the necessary support for further studies on the role of SIgA and IgG (including HCAbs) in Bactrian camel intestinal mucosal immunity.

## Materials and Methods

### Ethics statement

All experimental procedures were approved by the Animal Care and Use Committee (IACUC) of the College of Veterinary Medicine of Gansu Agricultural University (Approval No: GSAU-AEW-2013-0010). All efforts were made to minimize animal suffering.

### Experimental animals

Twenty-four clinically normal Alashan Bactrian camels were divided into the following four age groups: young (1–2 years, n = 6), pubertal (3–5 years, n = 6), middle-aged (6–16 years, n = 6) and old (17–18 years, n = 6). The animals were obtained from the Lejiawan slaughterhouse (Xining, Qinghai province of China) and were not starved before slaughter. The camels were intravenously anesthetized with sodium pentobarbital (20 mg/kg) and then euthanized via exsanguination to death.

### Microsection preparation and analysis

The abdomen of each Bactrian camel was cut open, and the whole small intestine, from the pylorus of the abomasum to the ileocecal aperture, was removed. Histological samples were obtained from the duodenum, jejunum and ileum. All samples were fixed in a 4% neutral paraformaldehyde solution for more than 15 days before sectioning. Paraffin sections were made using routine methods and were immunohistochemically analyzed following SABC staining.

### Primary antibodies

1. Rabbit polyclonal antibodies against Bactrian camels IgG were synthesized in our laboratory (Veterinary Pathology Laboratory of College of Veterinary Medicine, Gansu Agricultural University, China). The optimal working concentration of these primary antibodies was 1:1200 [32,33].
2. Rabbit polyclonal antibodies against Bactrian camel SIgA were synthesized in our laboratory (Veterinary Pathology Laboratory of College of Veterinary Medicine, Gansu Agricultural University, China). The optimal working concentration of these primary antibodies was 1:400 [34].

### Secondary antibodies

SABC goat anti-rabbit polyclonal antibodies were obtained from an immunohistochemical kit and were used according to the manufacturer’s instructions (Lot No. 07H3OCJ, Boster, Wuhan, Hubei, China).

### Light microscopy

In each group, the local distribution and characteristics of SIgA and IgG ASCs were carefully observed via light microscopy. In each segment of every sample, 30 sections were observed and photomicrographed using an Olympus DP-71 microscopy system.

### Statistical analysis

Five sections were randomly selected from each intestinal segment. In each section of the small intestinal LP, 10 microscopic fields were randomly selected, observed and photomicrographed. The number of SIgA ASCs and the number of IgG ASCs in each microscopic field were counted, and their respective densities were calculated (Image-Pro Plus 6.0). Statistically significant differences in the distribution densities between the two ASC populations in each intestinal segment of each group were determined via a one-way analysis of variance (ANOVA) followed by Duncan’s multiple range test. Data from both ASC populations within the same region were analyzed using Student's t-tests. Data analyses were performed using IBM SPSS V.21.0 (SPSS Inc., Chicago, USA). Differences were considered significant at *P*<0.05.

## Results

### Distribution characteristics of SIgA and IgG ASCs in the small intestines of Bactrian camels of different ages

**Fig 1:**
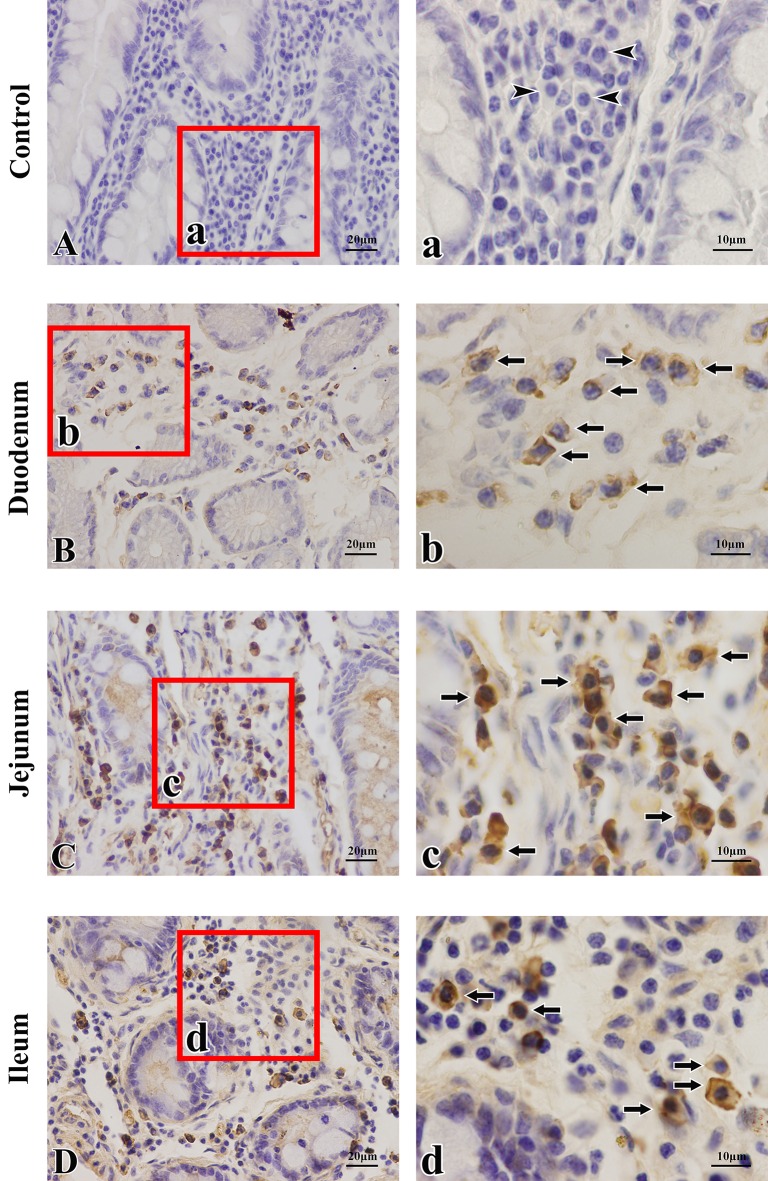
Immunohistochemical staining of SIgA antibody-secreting cells in the intestine of young Bactrian camels. **(A**) Negative control (lamina propria (LP) of the jejunum, with triangles indicating plasma cells). (**B**) Distribution of SIgA antibody-secreting cells (ASCs) in the duodenal LP. (**C**) Distribution of SIgA ASCs in the jejunal LP. (**D**) Distribution of SIgA ASCs in the ileal LP. SIgA ASCs were scattered in the small intestinal LP, and some cells aggregated around the crypts. Arrows indicate SIgA ASCs. Small pictures **(a-d)** are representative views from the four sublocations (original magnification: left column = 400×, right column = 1000×).

**Fig 2:**
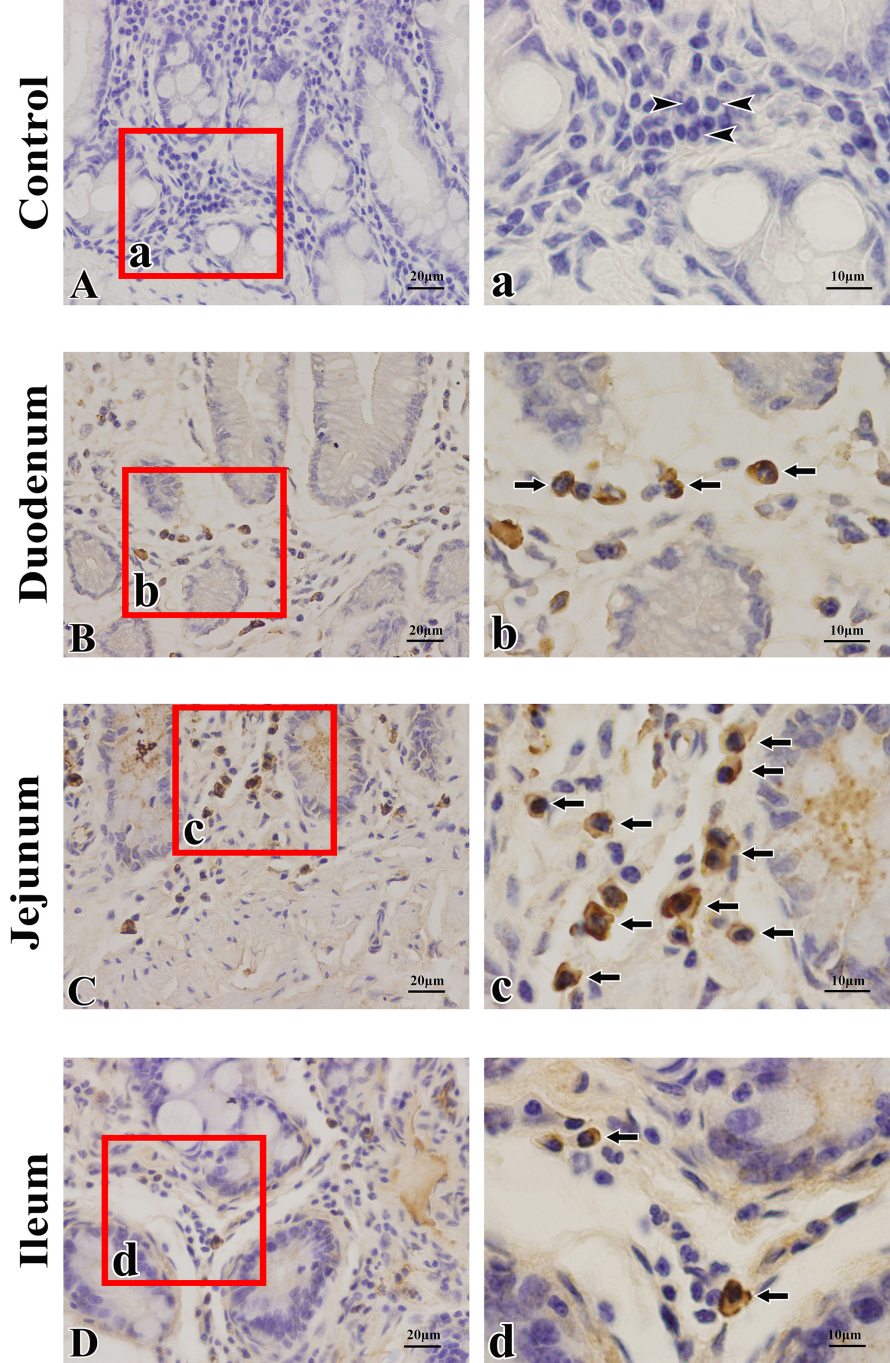
Immunohistochemical staining of IgG antibody-secreting cells in the small intestines of young Bactrian camels. (**A**) Negative control (lamina propria (LP) of the jejunum, with triangles indicating plasma cells). (**B**) Distribution of IgG antibody-secreting cells (ASCs) in the duodenal LP. (**C**) Distribution of IgG ASCs in the jejunal LP. (**D**) Distribution of IgG ASCs in the ileal LP. IgG ASCs were scattered in the LP of the small intestine, and some cells aggregated around the crypts. Arrows indicate IgG ASCs. Small pictures (**a-d**) are representative views from the four sublocations (original magnification: left column = 400×, right column = 1000×).

The distributions of SIgA ASCs in the duodenum, jejunum and ileum were similar in the young group. SIgA ASCs were scattered in the LP, and some of the cells aggregated around the intestinal crypt (Fig 1A–1D). In each segment of the small intestine from camels in the other groups, the distribution characteristics of SIgA ASCs were similar to those in the young group, and the distributions of IgG ASCs were similar to those of SIgA ASCs within the same region. IgG ASCs were scattered in the LP, and most of the cells aggregated around the intestinal crypts (Fig 2A–2D).

**Fig 3:**
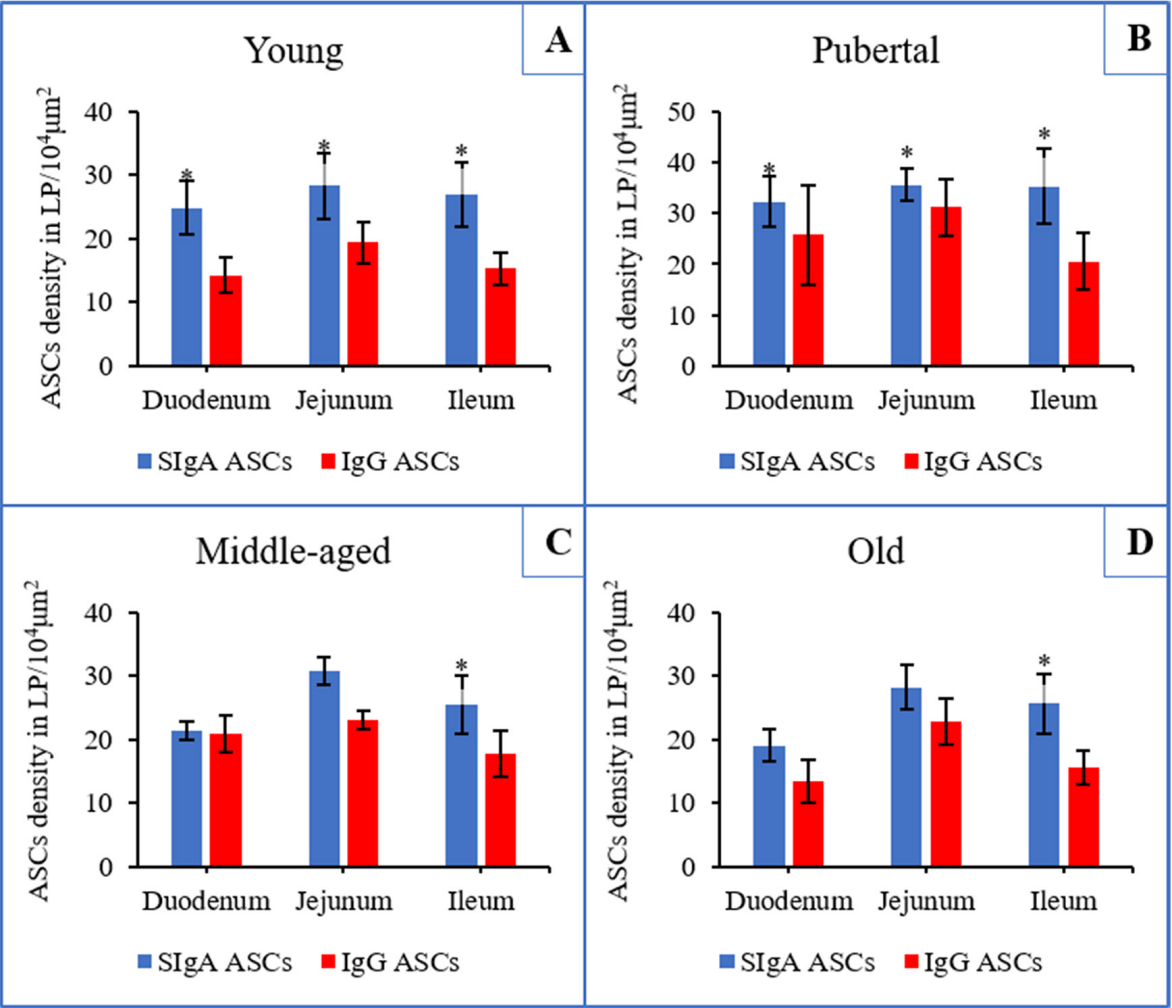
Distribution densities of SIgA and IgG antibody-secreting cells in the small intestine of Bactrian camels of different ages. (**A**), (**B**), (**C**), (**D**) indicate the young, pubertal, middle-aged and old groups, respectively. The distribution densities of SIgA antibody-secreting cells (ASCs) were higher than IgG ASCs within the same intestinal segment. *P<0.05 versus IgG ASCs.

In all groups, the statistical analysis of the results demonstrated that the distribution densities of SIgA ASCs in the LP were all higher than the densities of the IgG ASCs in the same locations. Among these age groups, the differences between the densities of the two types of ASCs were not significant in either the duodenum or jejunum of the middle-aged and old groups (*P*>0.05); however, there were significant differences between the densities of these two ASC populations in the duodenum, jejunum and ileum for all other groups (*P*<0.05) (Fig 3A–3D).

In all groups, the SIgA and IgG ASC distribution densities gradually increased from the duodenum to the jejunum and then decreased in the ileum (Fig 3A–3D).

### Changes in the SIgA and IgG ASCs distribution densities with age

**Fig 4:**
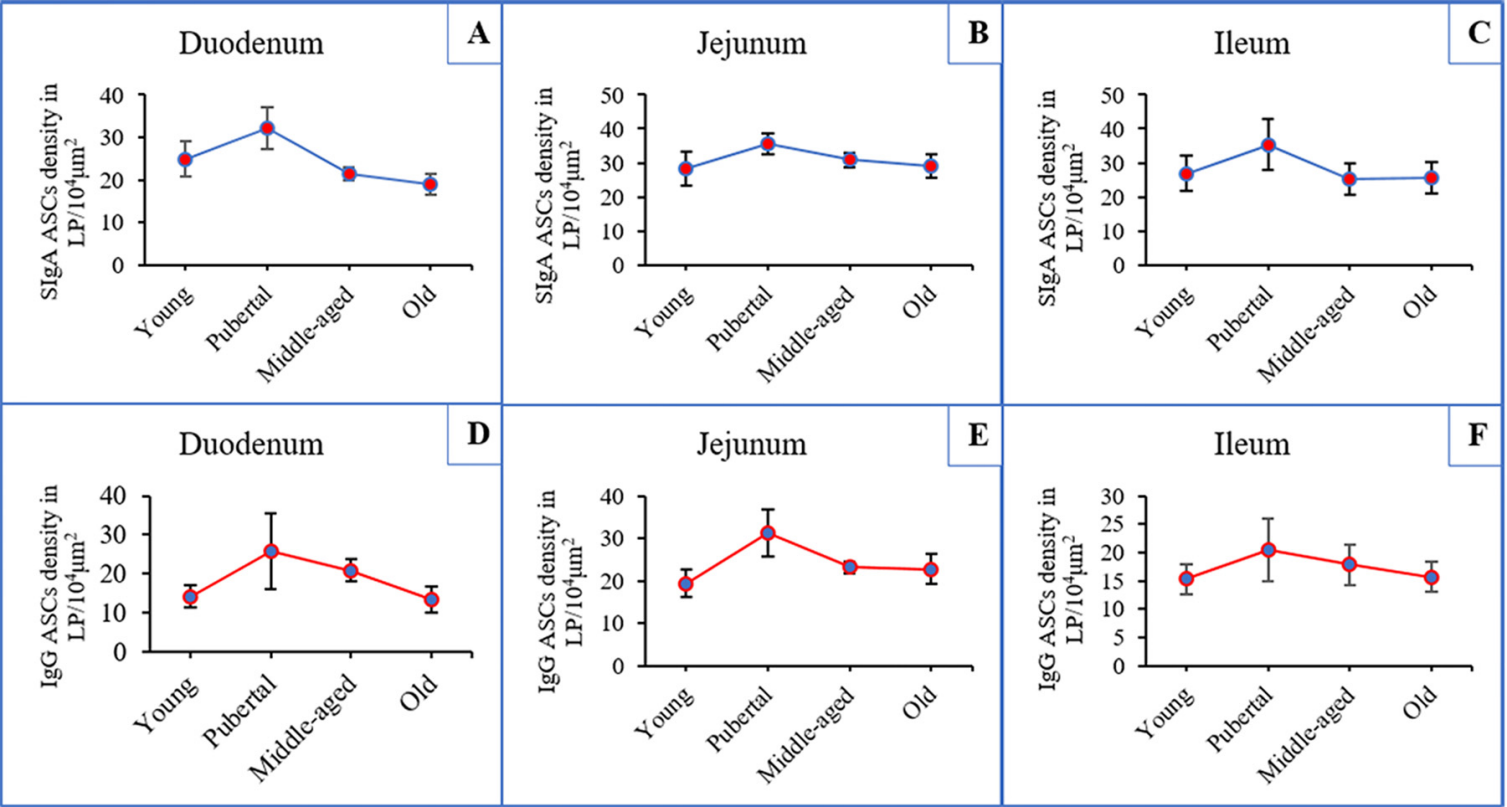
Age-related changes in SIgA and IgG antibody-secreting cell densities in different intestinal segments. (**A**), (**B**) and (**C**) indicate the age-related changes in the distribution densities of SIgA antibody-secreting cells (ASCs) in the duodenum, jejunum and ileum, respectively. (**D**), (**E**) and (**F**) indicate the age-related changes in the distribution densities of IgG ASCs in the duodenum, jejunum and ileum, respectively. Both ASC populations increased from the young to the pubertal group, peaked in the pubertal group, and gradually decreased with age.

In the duodenal LP, the distribution densities of SIgA ASCs gradually increased with increasing age from the young to the pubertal group, peaked in the pubertal group, and then decreased with age in the older groups (Fig 4A). These trends in the jejunum and the ileum were similar to those observed in the duodenum (Fig 4B and 4C). In the LP of the duodenum, jejunum and ileum, age-related changes in the distribution densities of IgG ASCs were similar to those of SIgA ASCs (Fig 4D–4F).

## Discussion

### Distribution characteristics of SIgA and IgG ASCs in the Bactrian camel small intestine

The observations in this study show that SIgA ASCs were scattered in the intestinal LP of Bactrian camels, and that some cells aggregated around the intestinal crypts. In addition, the distribution characteristics of IgG ASCs were similar to those of SIgA ASCs. These results suggest that the immune system can avoid the long-distance transport of immunoglobulins from ASCs to effector sites. This would significantly shorten the time required for SIgA and IgG to recognize invading pathogens, thus improving the sensitivity and accuracy of immunosurveillance and immune exclusion. This study also show that the scatted distribution characteristics are important for the ability of SIgA and IgG to form two protective immunoglobulin barriers in the intestine, which is critical because when protective immunoglobulin barriers are destroyed, the intestine is more vulnerable to gut pathogens [35–38].

The statistical analysis shows that the densities of SIgA and IgG ASCs were unevenly distributed in LP from the duodenum to the ileum in the small intestine of Bactrian camels. However, the SIgA and IgG ASCs distribution densities consistently increased from the duodenum to the jejunum and then decreased in the ileum, which is similar to the changes observed in other species and to the patterns of gut bacterial abundance observed in humans and other animals [39–41]. Moreover, several studies have indicated that gut microbiota can promote the development and maturation of the intestinal mucosal immune system [6,19,42–44]. However, in the present study, the ASC densities did not increase along with the increasing numbers of gut bacteria from the jejunum to the ileum. A possible reason for this is that many PPs are particularly distributed in the ileum, which are inductive sites for the intestinal mucosal immune system [45,46]. The protective layer of antibodies is maintained at an appropriate level in this area, which is beneficial for allowing the host to capture antigens and induce mucosal immune responses and tolerance. Hence, this distribution pattern of ASC populations might be closely related to the gut microbiota in different regions of the Bactrian camel intestine and to the functions of different intestinal regions in mucosal immunity.

In the same intestinal segment, the IgG ASC density was lower, sometimes significantly so, than the SIgA ASC density. This result is similar to those observed in the intestines of humans, rats and mice [21]. In addition, studies have indicated that huge amounts of SIgA are secreted and then transported to gut mucosal surfaces to form the first-line immunological barrier and regulate immune exclusion, immune tolerance and the intestinal microecology [12–18]. Therefore, IgG appears to provide a second line of defense. When the first-line immunologic barrier is destroyed, invasive pathogenic bacteria can cross the epithelial border, and IgG rapidly recruits innate immune cells, aiding in the elimination of invading bacteria [47]. Therefore, the distribution densities of these two types of ASCs provide evidence that SIgA and IgG can form two immunologic barriers in the small intestine of Bactrian camels.

### Changes in the SIgA and IgG ASCs distribution densities with age

The results show that the distribution characteristics of SIgA and IgG ASCs were the same in the small intestine of Bactrian camels at different ages, with both cell types scattered in the small intestinal LP. However, the densities of the two ASC populations increased from the young to the pubertal period, peaked at puberty, and then gradually decreased with age. This result is consistent with our previous studies on the aggregated lymphoid nodule area in the abomasum of Bactrian camels of different ages [24,25] and are also similar to the changes observed in peripheral blood IgA immunoblasts expressing intestinal homing molecules in rats of different ages [48]. Moreover, several studies have reported that SIgA ASCs, which only originate from gut-associated lymphoid tissue, home to intestinal LP from the peripheral blood via the actions of intestinal homing molecules [49,50]. Nevertheless, the differentiation, proliferation and maturation of B lymphocytes in the lymphoid follicles of aging animals have been shown to be inhibited, decreasing the number of effective plasma cells in the peripheral blood [51,52]. Hence, these results provide support for the further study of mucosal immunosenescence in the digestive tract of Bactrian camels.

## Conclusions

This study demonstrates that Bactrian camel SIgA and IgG ASCs were both diffusely distributed in the mucosal LP of the small intestine and the distribution densities of SIgA ASCs were all higher than those of IgG ASCs within the same regions across the age groups. The distribution densities of SIgA and IgG ASCs increased from the duodenum to the jejunum and then decreased in the ileum within each age group. Both ASC populations gradually increased with age, peaked during the pubertal period, and subsequently declined with age. The scattered distribution patterns observed here provide a rationale for further researching on why and how SIgA and IgG form two fully-protective immunoglobulin barriers in the intact intestine, as well as on the progression of gut mucosal immunosenescence, in Bactrian camels. In addition, this study establishes a foundation for further research on the relationships between SIgA, IgG and the gut microbiota in the small intestine of Bactrian camels.
